# Photobiomodulation outperforms ultrasound in reducing IL-1***β***-driven chondrocyte inflammation

**DOI:** 10.1063/5.0323327

**Published:** 2026-06-09

**Authors:** Sofia Oliveira, Débora Ferreira, Ligia R. Rodrigues, Filipe S. Silva, Betina B. Hinckel, Óscar Carvalho, Ana Leal

**Affiliations:** 1 CMEMS—UMinho, University of Minho, 4800-058 Guimarães, Portugal; 2 CEB—Centre of Biological Engineering, University of Minho, Campus de Gualtar, 4710-057 Braga, Portugal; 3 LABBELS—Associate Laboratory, 4710-057 Braga, Guimarães, Portugal; 4 Department of Orthopaedic Surgery, William Beaumont Hospital, Royal Oak, Michigan 48073, USA; 5 Dom Henrique Research Centre, Porto, Portugal

## Abstract

Therapeutic ultrasound (US) and photobiomodulation (PBM) are promising treatment modalities for restoring articular cartilage. Owing to their different mechanisms of action, individual or combined stimulation may elicit different bioeffects on chondrocytes. This study aimed to evaluate the potential of US and PBM, applied alone or combined, in augmenting chondrocytes' responses toward cartilage matrix synthesis and reverting their catabolic activities, with or without interleukin-1β (IL-1β) incubation. Human chondrocytes were cultured under basal or proinflammatory conditions (1 ng/ml IL-1β) and stimulated daily for 6 days with US, PBM, US followed by PBM, and PBM followed by US. Chondrocyte activity, along with protein and mRNA expression of cartilage synthesis- and degradation-related markers, was analyzed. IL-1β incubation did not significantly affect chondrocytes' metabolic activity or sulfated glycosaminoglycans (GAGs) production. Both US and PBM, alone or combined, increased the protein and mRNA of collagen type II (*COL II*) and aggrecan (*ACAN*) under basal and proinflammatory conditions. PBM had a stimulatory effect on GAG synthesis. While PBM did not influence the expression of cartilage destruction markers, US potentiated the chondrocytes' response to inflammation by increasing metalloproteinases and *IL-1β* mRNA levels. The sequential combination of US and PBM induced distinct bioeffects depending on the application order, but these were not superior to the effects of individual treatments. As this study used chondrocytes from a single donor, generalizability may be limited. While both US and PBM stimulated matrix synthesis, PBM showed a comparatively greater ability to mitigate chondrocyte degradation and may be of interest for cartilage repair strategies.

## INTRODUCTION

Articular cartilage is the bearing surface of synovial joints and is constantly subjected to mechanical loading. Chondrocytes, the resident cells, sense mechanical deformation and respond by maintaining a healthy balance between the synthesis and degradation of the cartilage matrix. This matrix is mainly composed of collagen type II (COL II) fibers and proteoglycans, whose interplay contributes to their remarkable mechanical performance.^1,2^ However, excessive mechanical loading may lead to cartilage degeneration.^3–5^

Upon mechanical injury, the homeostasis of articular cartilage is altered. The tissue exhibits surface fibrillation, crushing, and fissures,^3,6^ resulting in the breakdown of the collagen fibrillar network and the depletion of proteoglycans.^7,8^ At the cellular level, apoptosis can occur, leading to cell death.^9^ The remaining viable chondrocytes produce proinflammatory cytokines, such as interleukin-1β (IL-1β) and tumor necrosis factor (TNF-α), which in turn activate the production of matrix-degrading proteases [e.g., metalloproteinases (MMP)-1, -2, -3, -13] to degrade the matrix, leading to cartilage loss.^10–13^

Therapeutic ultrasound (US) and photobiomodulation (PBM) are noninvasive treatment options that have attracted increasing attention within the scientific community. US is a form of acoustic mechanical energy oscillating at frequencies above human audibility (>20 kHz), while PBM involves the application of light in the visible and near-infrared spectrum (600–1100 nm).^14,15^ Current evidence suggests that both US and PBM hold promise for the restoration of injured articular cartilage due to their potential effects on cartilage regeneration, pain relief, inflammation reduction, and improved physical function. At the cellular level, US induces mechanical vibrations on the cell surface, triggering chondrocytes' mechanoreceptors, such as ion channels and integrins.^15–19^ Meanwhile, light used in PBM is absorbed by mitochondria but can also activate light-sensitive ion channels.^20^ Following stimulation, the activation of chondrocyte receptors elicits intracellular signaling pathways that lead to biological responses, including enhanced metabolism, cell proliferation, or protein synthesis.^21–23^

Although both US and PBM have shown promising effects on chondrocyte activity and cartilage regeneration, most studies have investigated these modalities independently. Considering that US primarily acts through mechanical mechanotransduction pathways, while PBM mainly modulates mitochondrial activity and cellular metabolism, their combination may elicit complementary or even synergistic biological responses. However, studies exploring the combined effects of US and PBM on chondrocytes, particularly under inflammatory conditions relevant to cartilage degeneration, remain scarce. By leveraging their distinct mechanisms of action, the combined application of US and PBM represents an intriguing approach to potentially amplify these biological effects. To the best of our knowledge, this approach has not yet been investigated in the context of chondrocyte inflammation, highlighting the novelty and relevance of the present study to the current literature.

The aim of this study is to evaluate the effects of US and PBM, applied individually and sequentially in combination, on human chondrocytes, with a particular focus on their potential to counteract degeneration-related events, namely, inflammation. To this end, human chondrocytes were cultured under either basal or proinflammatory conditions and subsequently stimulated daily for 6 days with US, PBM, or both. The impact of these treatments was investigated by assessing cell activity, as well as the synthesis and degradation of cartilage matrix components, in both IL-1β-free and IL-1β-treated chondrocytes, to clarify the influence of these stimuli, individually or combined, in restoring degenerated articular cartilage.

## RESULTS

### Effects on chondrocyte metabolic activity

**FIG. 1.**
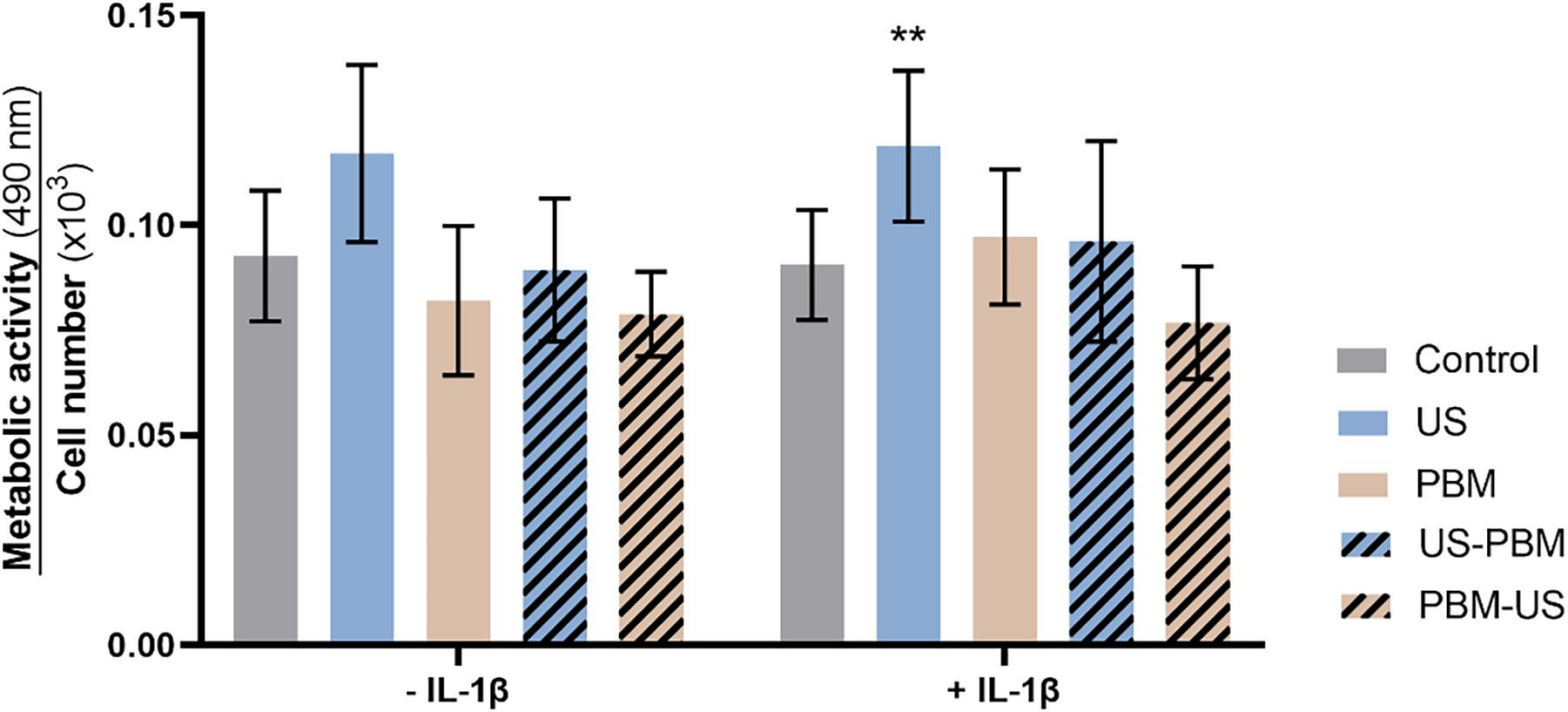
Metabolic activity of non-stimulated (control) and stimulated human chondrocytes with only ultrasound (US), only photobiomodulation (PBM), US followed by PBM (US-PBM), and PBM followed by US (PBM-US), with and without incubation with 1 ng/ml IL-1β (n = 3 independent experiments). Stimulation was performed daily for 6 days with the following parameters: US at 2.00 MHz, 250 mW/cm^2^, pulsed mode at 1 Hz and 50% duty cycle, for 20 min; and PBM with 940 nm at 17 mW/cm^2^, continuous mode for 3 min. One-way ANOVA followed by Tukey's *post hoc* test was used to determine statistical differences compared to non-stimulated chondrocytes, with significance denoted as ^**^p < 0.01.

The metabolic activity, normalized by cell number, of IL-1β-free and IL-1β-treated chondrocytes was assessed after 6 days of daily stimulation (Fig. 1).

The normalized metabolic activity was significantly increased compared to non-stimulated cells following US only in IL-1β-treated chondrocytes (p = 0.011). The remaining stimulation modalities did not affect the chondrocytes' metabolic activity. Additionally, the IL-1β incubation had no significant impact on chondrocyte activity, as similar normalized metabolic activity was observed with and without IL-1β treatment.

### Effects on cartilage synthesis and dedifferentiation

After daily stimulation with US and PBM, either alone or in combination, the synthesis of COL II, ACAN, and COL I was investigated.

#### Influence on extracellular matrix synthesis

**FIG. 2.**
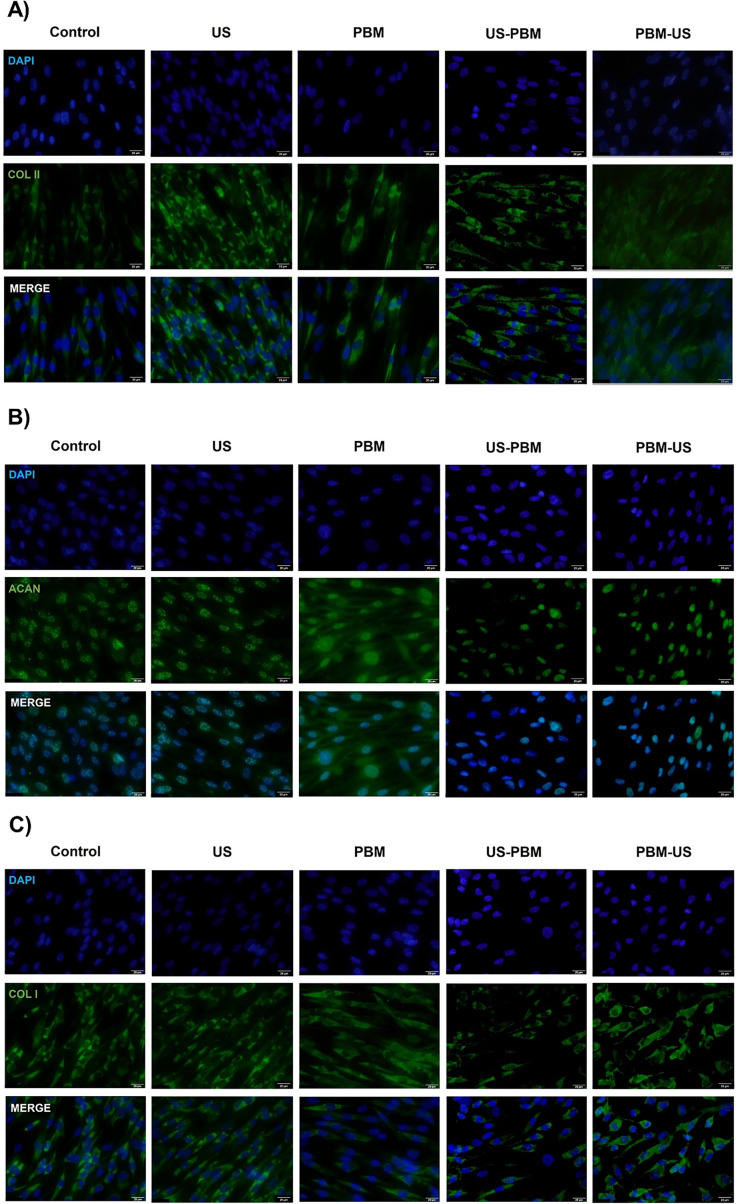
Representative fluorescence microscopy images of non-stimulated (control) and stimulated human chondrocytes treated with only ultrasound (US), only photobiomodulation (PBM), US followed by PBM (US-PBM), and PBM followed by US (PBM-US), without IL-1β incubation (n = 3 independent experiments). Stimulation was conducted daily for 6 days with the following parameters: US at 2.00 MHz, 250 mW/cm^2^, pulsed mode at 1 Hz and 50% duty cycle, for 20 min; and PBM with 940 nm at 17 mW/cm^2^, continuous mode for 3 min. The cell nucleus was stained blue with DAPI, and (a) COL II, (b) ACAN, and (c) COL I expressions were stained green. Scale bar: 20 *μ*m.

**FIG. 3.**
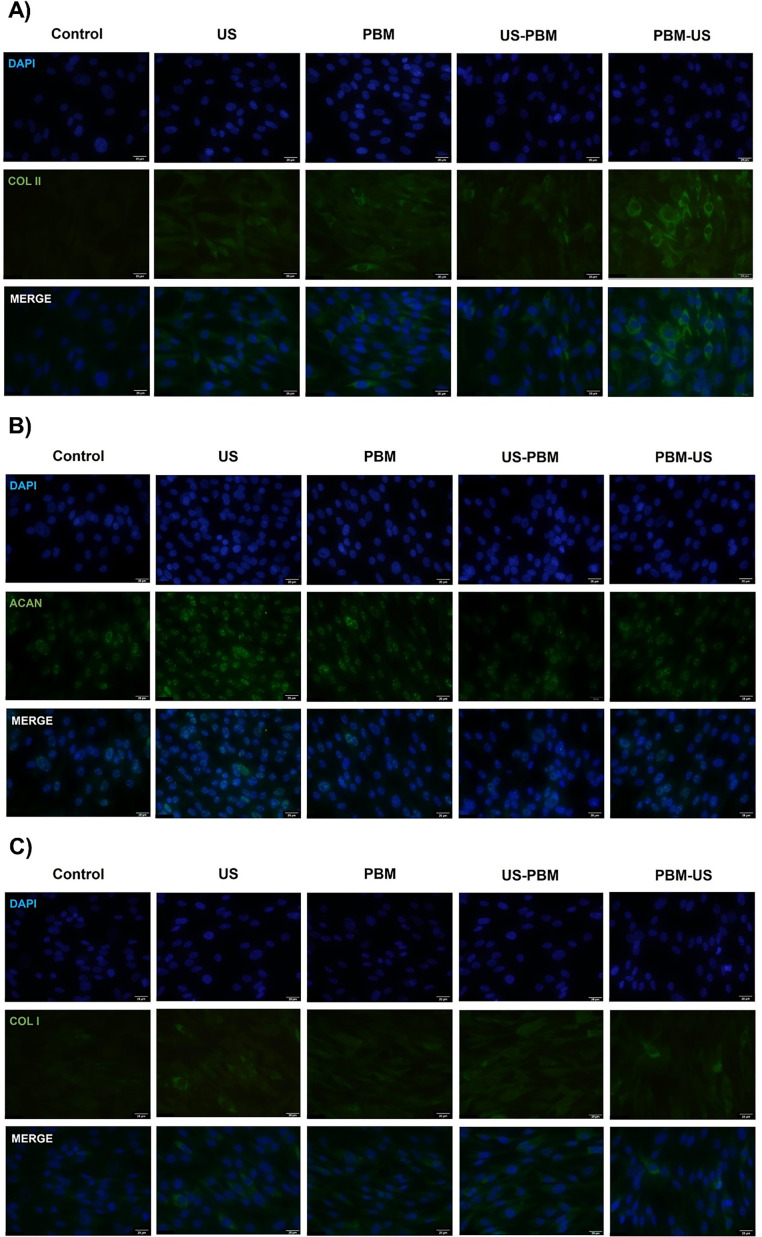
Representative fluorescence microscopy images of non-stimulated (control) and stimulated human chondrocytes with only ultrasound (US), only photobiomodulation (PBM), US followed by PBM (US-PBM), and PBM followed by US (PBM-US) with 1 ng/ml IL-1β incubation (n = 3 independent experiments). Stimulation was conducted daily for 6 days with the following parameters: US at 2.00 MHz, 250 mW/cm^2^, pulsed mode at 1 Hz and 50% duty cycle, for 20 min; and PBM with 940 nm at 17 mW/cm^2^, continuous mode for 3 min. The cell nucleus was stained blue with DAPI, and (a) COL II, (b) ACAN, and (c) COL I expressions were stained green. Scale bar: 20 *μ*m.

Immunocytochemistry was conducted in both IL-1β-free (Fig. 2) and IL-1β-treated chondrocytes (Fig. 3).

Fluorescence microscopy images revealed that chondrocytes, without IL-1β incubation, were positively stained for COL II [Fig. 2(a)], ACAN [Fig. 2(b)], and COL I [Fig. 2(c)]. In comparison to the non-stimulated group, the deposition of COL II and ACAN in the chondrocytes' matrix was similar across all stimulation groups. Both stimulated and non-stimulated chondrocytes expressed COL I, a dedifferentiation marker.

After IL-1β incubation, COL II [Fig. 3(a)], ACAN [Fig. 3(b)], and COL I [Fig. 3(c)] expression was less prominent compared to the same protein expressions in IL-1β-free chondrocytes (Fig. 2). The staining intensity for ACAN was similar across the stimulation groups. Regardless of the stimulation group, a higher deposition of COL I in the chondrocyte matrix was detected compared to the control.

#### Influence on sulfated glycosaminoglycan production

**FIG. 4.**
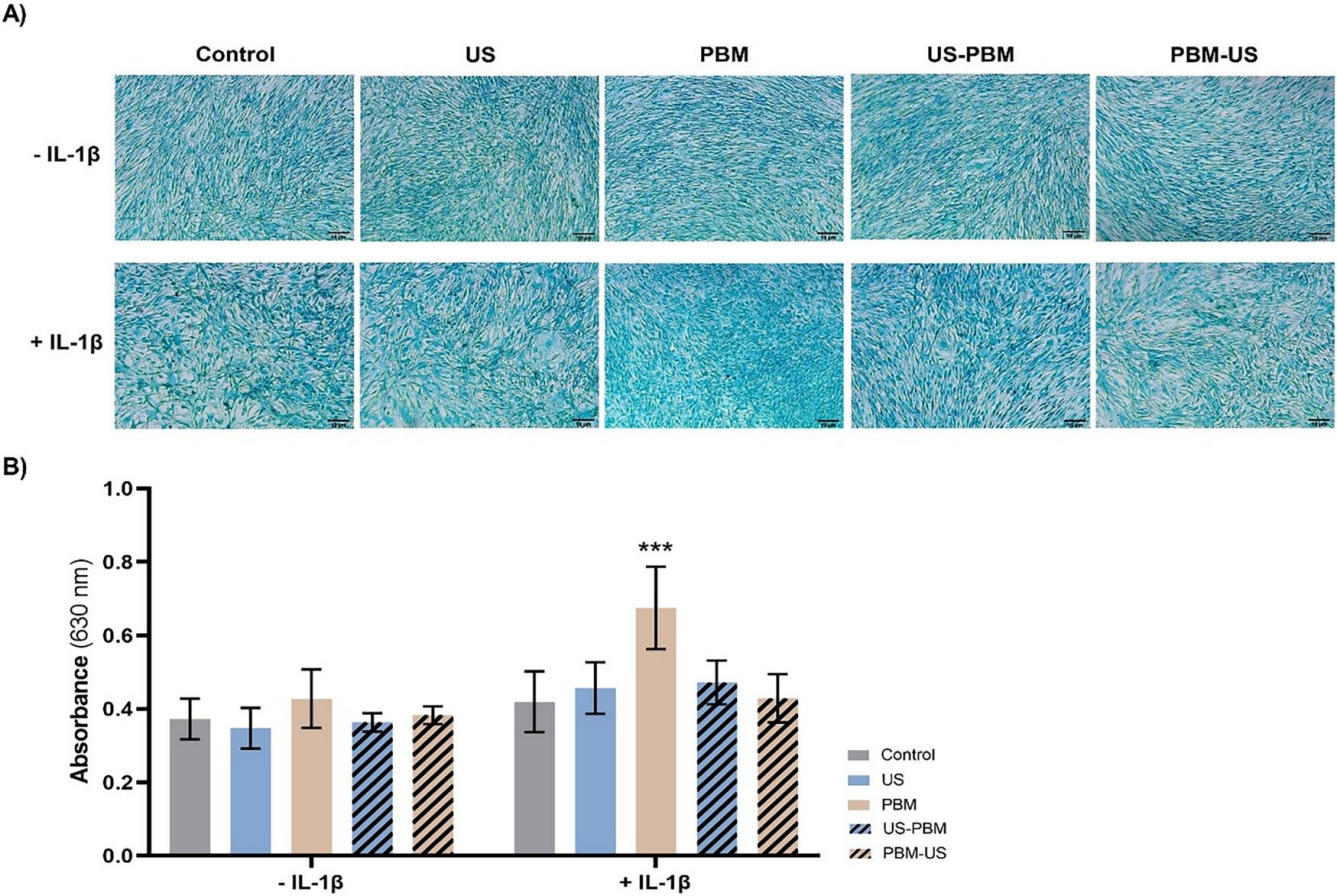
(a) Representative microscopy images of non-stimulated (control) and stimulated human chondrocytes with only ultrasound (US), only photobiomodulation (PBM), US followed by PBM (US-PBM), and PBM followed by US (PBM-US) with and without 1 ng/ml IL-1β incubation, stained with alcian blue. (b) Quantification of alcian blue staining (n = 3 independent experiments). Stimulation was conducted daily for 6 days with the following parameters: US at 2.00 MHz, 250 mW/cm^2^, pulsed mode at 1 Hz and 50% duty cycle, for 20 min; and PBM with 940 nm at 17 mW/cm^2^, continuous mode for 3 min. Scale bar: 10 *μ*m. Kruskal–Wallis followed by Tukey's *post hoc* test was used to determine statistical differences compared to non-stimulated chondrocytes, with significance denoted as ^***^p < 0.001.

The synthesis of sulfated glycosaminoglycans (GAGs) following stimulation, with and without IL-1β incubation, was assessed by staining with alcian blue [Fig. 4(a)] and quantifying the absorbance of the extracted alcian blue [Fig. 4(b)].

Microscopy images revealed that both IL-1β-free and IL-1β-treated chondrocyte cultures were positively stained for sulfated GAGs, as indicated by the alcian blue staining [Fig. 4(a)]. The IL-1β incubation did not affect the expression of GAGs, as similar quantification of eluted alcian blue was observed with and without IL-1β treatment. In IL-1β-free chondrocytes, the quantification of eluted alcian blue showed no differences among stimulation modalities in comparison to non-stimulated chondrocytes [Fig. 4(b)]. However, after IL-1β incubation, chondrocytes exposed only to PBM significantly increased the production and deposition of GAGs (p < 0.001) compared to non-stimulated cells. This finding is consistent with the microscopy images, which show a more intense staining in chondrocytes stimulated with US-PBM or PBM only.

#### Influence on protein content of extracellular matrix proteins

**FIG. 5.**
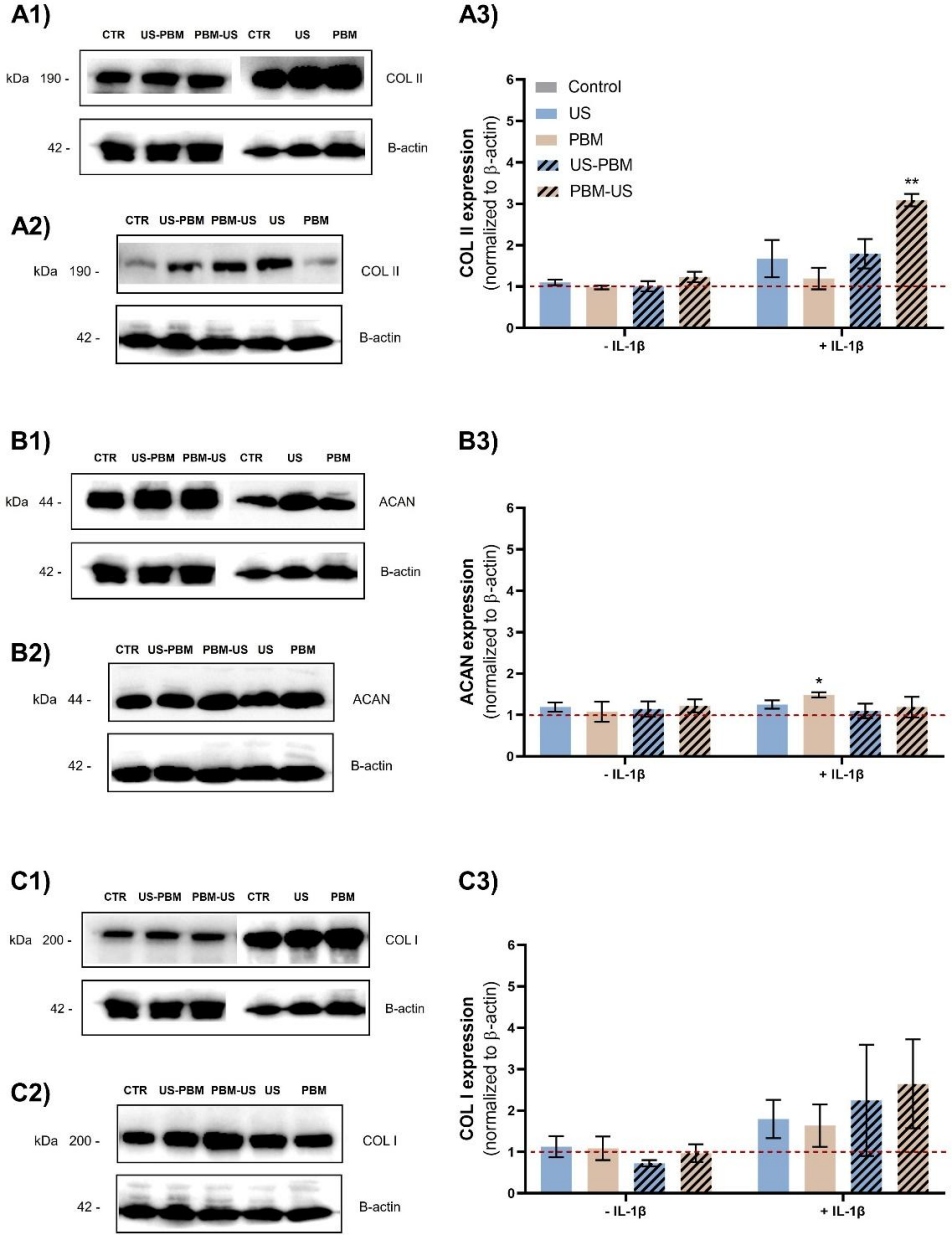
Representative images of western blot analysis of (a) COL II, (b) ACAN, and (c) COL I expression in (a1), (b1), and (c1) IL-1β-free chondrocytes and in (a2), (b2), and (c2) IL-1β-treated chondrocytes. Western blot analysis of protein expression for (a3) COL II, (b3) ACAN, and (c3) COL I in non-stimulated (control) and stimulated human chondrocytes with only ultrasound (US), only photobiomodulation (PBM), US followed by PBM (US-PBM), and PBM followed by US (PBM-US), with and without incubation with 1 ng/ml IL-1β (n = 2 independent experiments). Stimulation was conducted daily for 6 days with the following parameters: US at 2.00 MHz, 250 mW/cm^2^, pulsed mode at 1 Hz and 50% duty cycle, for 20 min; and PBM with 940 nm at 17 mW/cm^2^, continuous mode for 3 min. One-way ANOVA followed by Tukey's *post hoc* test was used to determine statistical differences compared to non-stimulated chondrocytes, with significance denoted as ^*^p < 0.05 and ^**^p < 0.01. Uncropped western blot images can be found in supplementary material Fig. S1.

Western blot analysis was conducted to determine the protein content of IL-1β-free and IL-1β-treated chondrocyte cultures (Fig. 5). The protein level of COL II produced by human IL-1β-free chondrocytes remained unchanged after stimulation [Fig. 5(a)]. However, after IL-1β incubation, chondrocytes exposed to PBM-US increased the synthesis of COL II by threefold (p = 0.004) compared to control, while the expression remained unchanged in the other stimulation groups.

In the IL-1β-free chondrocytes, ACAN production was unaffected following stimulation. In contrast, the individual application of PBM significantly elevated ACAN synthesis by 1.5-fold (p = 0.014) when compared to non-stimulated cells. The combination of both stimuli did not alter ACAN expression [Fig. 5(b)].

Neither individual nor combined stimulation influenced the protein level of COL I in IL-1β-free chondrocytes, as its expression remained unaltered. Although there was a trend toward increased COL I expression following stimulation, COL I levels remained unchanged in IL-1β-treated chondrocytes [Fig. 5(c)].

#### Influence on gene expression of extracellular matrix proteins

**FIG. 6.**
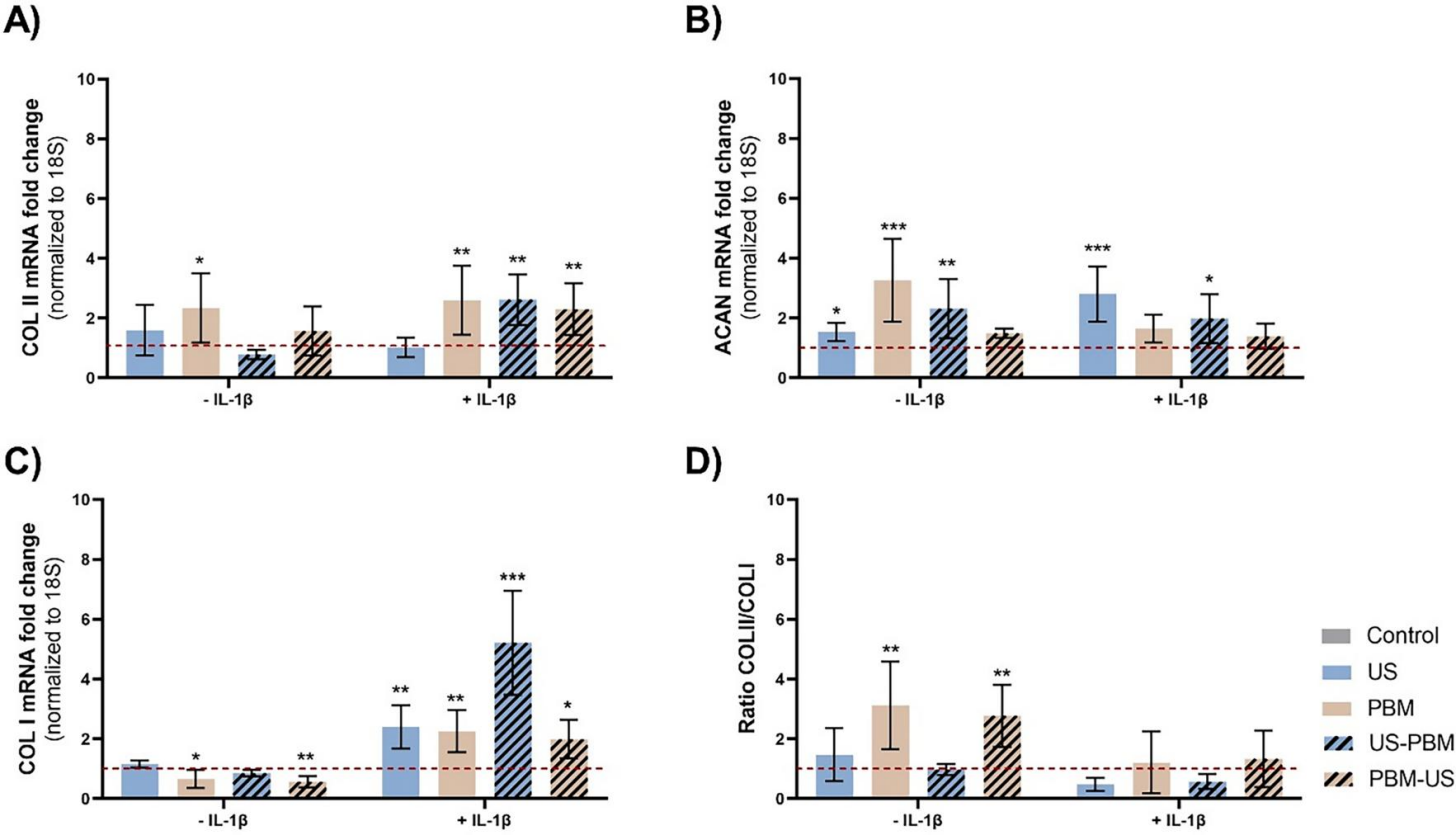
Quantification of mRNA levels for (a) *COL II*, (b) *ACAN*, and (c) *COL I* in non-stimulated (control) and stimulated human chondrocytes with only ultrasound (US), only photobiomodulation (PBM), US followed by PBM (US-PBM), and PBM followed by US (PBM-US), with and without incubation with 1 ng/ml IL-1β (n = 2 independent experiments). Ratio of COL II/COL I **(**d). Stimulation was conducted daily for 6 days with the following parameters: US at 2.00 MHz, 250 mW/cm^2^, pulsed mode at 1 Hz and 50% duty cycle, for 20 min; and PBM with 940 nm at 17 mW/cm^2^, continuous mode for 3 min. Kruskal–Wallis followed by Tukey's *post hoc* test was used to determine statistical differences for *COL II* and *ACAN*, and one-way ANOVA followed by Tukey's *post hoc* test was used to determine statistical differences for *COL I* compared to non-stimulated chondrocytes, with significance denoted as ^*^p < 0.05, ^**^p < 0.01, and ^***^p < 0.001.

The gene expression of extracellular matrix (ECM) components, *COL II*, *ACAN*, and *COL I*, was investigated by quantitative PCR (qPCR) for IL-1β-free and IL-1β-treated chondrocytes following stimulation (Fig. 6).

The mRNA level of *COL II* [Fig. 6(a)] enhanced by 2.3-fold (p = 0.034) on IL-1β-free chondrocytes stimulated with PBM alone. However, stimulation reversed the IL-1β-induced effects in IL-1β-treated chondrocyte cultures, with *COL II* mRNA expression elevated by 2.6-fold after PBM alone (p = 0.001) or US-PBM (p = 0.001), and by 2.3-fold after PBM-US (p = 0.006).

The mRNA level of *ACAN* [Fig. 6(b)] was consistently and significantly superior in IL-1β-free chondrocytes subjected to stimulation, regardless of the modality, with increases ranging from 1.5- to 3.3-fold compared to control (p < 0.01), except for PBM-US stimulation. After IL-1β treatment, only US alone (p < 0.001) and US-PBM (p = 0.013) significantly enhanced *ACAN* mRNA expression by 2.8- and 2.0-fold, respectively, compared to non-stimulated chondrocytes. No significant changes in *ACAN* mRNA expression were observed when PBM was applied to IL-1β-treated chondrocytes, either alone or combined with US.

The mRNA expression of *COL I* [Fig. 6(c)] significantly decreased in IL-1β-free chondrocytes by 0.7-fold after PBM alone (p = 0.026) and 0.6-fold after PBM-US (p = 0.003), compared to non-stimulated cells. Nevertheless, after treating chondrocytes with IL-1β, no stimulation modality was effective in reducing *COL I* mRNA expression, as it significantly increased by 2.0- to 5.2-fold in comparison to control.

The COL II/COL I mRNA expression ratio significantly increased in IL-1β-free chondrocytes stimulated with PBM alone (p = 0.002) and PBM-US (p =0.006). In the remaining stimulation conditions, the ratio remained comparable to the control group. Following IL-1β treatment, the COL II/COL I ratio did not show significant differences among experimental groups. However, a downward trend was observed in chondrocytes stimulated with US alone and US-PBM.

### Effects on cartilage degradation

#### Influence on MMP-13, IL-1β, and MMP-3 synthesis

**FIG. 7.**
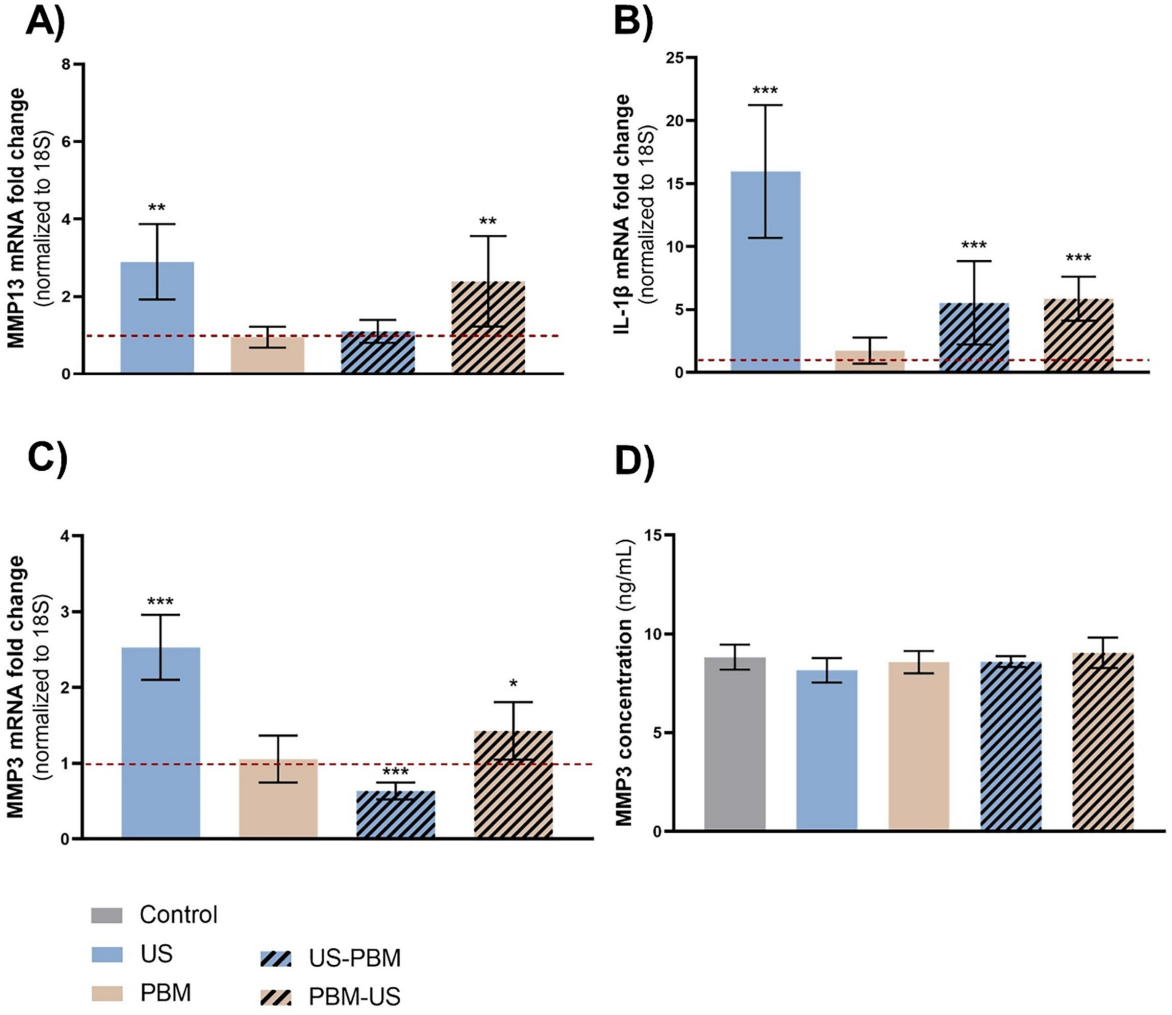
Quantification of mRNA levels for (a) *MMP-13*, (b) *IL-1β*, (c) *MMP-3*, and soluble MMP-3 expression in non-stimulated (control) and stimulated human chondrocytes with only ultrasound (US), only photobiomodulation (PBM), US followed by PBM (US-PBM), and PBM followed by US (PBM-US) with incubation with 1 ng/ml IL-1β (n = 3 independent experiments). Stimulation was conducted daily for 6 days with the following parameters: US at 2.00 MHz, 250 mW/cm^2^, pulsed mode at 1 Hz and 50% duty cycle, for 20 min; and PBM with 940 nm at 17 mW/cm^2^, continuous mode for 3 min. Kruskal–Wallis, followed by Tukey's *post hoc* test, was used to determine statistical differences for *MMP-13* mRNA expression and one-way ANOVA followed by Tukey's *post hoc* test was used to determine statistical differences for *IL-1β* and *MMP-3* expressions compared to non-stimulated chondrocytes, with significance denoted as ^*^p < 0.05, ^**^p < 0.01, and ^***^p < 0.001.

The mRNA levels of *MMP-13*, *IL-1β*, and *MMP-3* following stimulation on IL-1β-treated chondrocytes were investigated (Fig. 7). Stimulating IL-1β-treated chondrocytes with US-PBM did not alter *MMP-13* mRNA expression. However, applying US alone promoted a significant increase in *MMP-13* expression by 2.9-fold (p = 0.002). The influence of US was also marked in IL-1β-treated chondrocytes exposed to PBM-US, which exhibited a significantly enhanced *MMP-13* mRNA expression by 2.4-fold (p = 0.002), as opposed to PBM alone, which did not change *MMP-13* mRNA levels [Fig. 7(a)].

The application of PBM alone did not influence the *IL-1β* mRNA expression produced by IL-1β-treated chondrocytes. In contrast, combined stimulation significantly increased *IL-1β* expression by 5.5- to 5.8-fold (p < 0.001). *IL-1β* mRNA expression was remarkably potentiated following US alone, with a 16-fold increase (p < 0.001) compared to control [Fig. 7(b)].

The mRNA expression of *MMP-3* followed a similar pattern to *MMP-13*, since both PBM-US (p = 0.017) and US alone (p < 0.001) significantly increased *MMP-3* mRNA levels by 1.4- and 2.5-fold, respectively, while PBM alone did not alter this outcome [Fig. 7(c)]. Nevertheless, IL-1β-treated chondrocytes subjected to combined stimulation of US-PBM significantly reduced the expression of *MMP-3* by 0.6-fold (p < 0.001). An enzyme-linked immunosorbent assay (ELISA) was conducted to investigate the concentration of MMP-3 secreted to the culture medium by IL-1β-treated chondrocytes, but no differences were found in comparison to non-stimulated cells [Fig. 7(d)].

#### Influence on MMP-2 activity

**FIG. 8.**
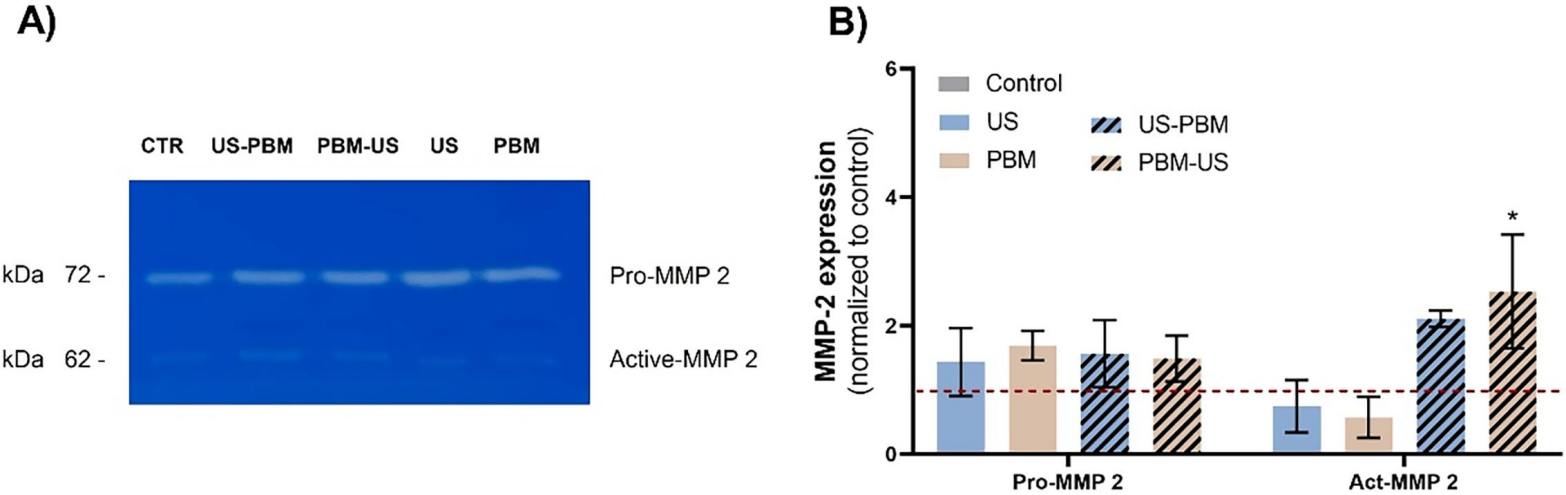
(a) Representative images of gelatin zymography and (b) gelatin zymography analysis of pro-MMP-2 and active-MMP-2 expression in non-stimulated (control) and stimulated human chondrocytes with only ultrasound (US), only photobiomodulation (PBM), US followed by PBM (US-PBM), and PBM followed by US (PBM-US) with incubation with 1 ng/ml IL-1β (n = 3 independent experiments). Stimulation was conducted daily for 6 days with the following parameters: US at 2.00 MHz, 250 mW/cm^2^, pulsed mode at 1 Hz and 50% duty cycle, for 20 min; and PBM with 940 nm at 17 mW/cm^2^, continuous mode for 3 min. One-way ANOVA followed by Tukey's *post hoc* test was used to determine statistical differences compared to non-stimulated chondrocytes, with significance denoted as ^*^p < 0.05.

Gelatin zymography was used to assess the expression of MMP-2 secreted by IL-1β-treated chondrocytes into the culture medium (Fig. 8).

For proteolytic cleavage of the cartilage matrix to occur, MMP-2 must be activated after its production, and thus, both pro- and active forms of MMP-2 were analyzed. The presence of pro-MMP-2 in IL-1β-treated chondrocytes was similar across all stimulation modalities. The active form of MMP-2 appeared in all stimulation conditions, but the combined application of PBM and US significantly increased its expression by 2.5-fold (p = 0.015). The individual application of US and PBM did not influence the active form of MMP-2 compared to non-stimulated cells.

## DISCUSSION

This study demonstrated that the combination of US and PBM did not produce additive or synergistic effects. Notably, PBM promoted anabolic activity with minimal catabolic activation, whereas US enhanced inflammatory responses. These findings suggest that, although both modalities are biologically active, PBM may be a more favorable candidate for cartilage repair strategies.

Inflammation is one of the earliest events that takes place upon cartilage injury. IL-1β induces concentration-dependent inflammatory responses, with higher doses strongly promoting catabolic activity and matrix degradation.^24–27^ In contrast, 1 ng/ml is considered a biologically relevant threshold that modulates chondrocyte activity without inducing cytotoxicity.^28^ Therefore, chondrocytes were incubated with 1 ng/ml IL-1β to establish an *in vitro* model of early-stage degeneration. In line with previous reports, IL-1β did not affect cell viability.^29–32^ Although limited effects were observed at protein and metabolic levels, a moderate increase in TNF-α gene expression was detected (supplementary material Fig. S2), indicating an inflammatory response. Also, IL-1β-treated chondrocytes showed reduced COL II and ACAN immunostaining compared with untreated cells, consistent with suppression of cartilage matrix protein expression.

Under basal conditions, stimulation affected cartilage marker gene expression but not protein levels, indicating a disconnect between transcriptional and translational responses. This may reflect post-transcriptional regulation, including activation of translational pathways (AKT, ERK, and mTOR), differences in translational efficiency, protein stability, and mechanosensitive RNA regulation.^33,34^ These findings highlight regulation beyond transcript levels and support integrating transcriptomic and proteomic analyses to capture mechanostimulation responses. Under proinflammatory conditions, BM consistently enhanced cartilage matrix gene and protein expression, counteracting IL-1β-induced catabolic effects, in agreement with previous studies.^18,35–38^ US increased ACAN gene expression but not other matrix components, with divergent reports likely related to IL-1β concentration (10 vs <2.5 ng/ml).^31,32,39^

COL I expression was assessed to evaluate stimulation effects on chondrocyte dedifferentiation in monolayer culture. Neither PBM nor US prevented IL-1β-induced dedifferentiation, as shown by increased COL I expression and a reduced COL II/COL I ratio under inflammatory conditions, in agreement with previous reports.^40^ Although PBM improved the COL II/COL I ratio under basal conditions, this effect was not maintained under proinflammatory conditions, suggesting limited ability to preserve the chondrocyte phenotype in degenerative environments.

MMPs play an important role in the degradation of the cartilage matrix. First, collagenases (MMP-3 and MMP-13) cleave collagen fibers and denature collagen fibrils, after which gelatinases, such as MMP-2, digest these denatured fibers.^11^ In this study, US stimulation increased *IL-1β*, *MMP-3*, and *MMP-13* mRNA expression, which may limit its standalone therapeutic potential. PBM alone did not significantly alter inflammatory markers but modulated US-induced effects in a sequence-dependent manner. When PBM was applied before US, chondrocytes showed increased *MMP-3* and *MMP-13* mRNA expression. However, when the order was reversed (i.e., US first and PBM after), *MMP-3* mRNA expression was significantly reduced. These findings suggest a potential modulatory role of PBM with significant implications for therapeutic scheduling. In contrast to our results, previous studies reported reduced *MMP-13* expression following US,^32,39^ likely reflecting differences in timing. While those studies measured gene expression within 6 h post-stimulation, we assessed *MMP* levels 24 h after the last session. In fact, one study showed that the inhibitory effect on *MMP-13* was no longer detectable 1 h post-stimulation.^24^ Although PBM has been reported to decrease the expression of inflammatory markers *in vivo*,^41–43^ no significant effects were observed on *IL-1β*, *MMP-3*, and *MMP-13* mRNA expression. This contrasts with one study that reported reduced *IL-1β* and *MMP-3* levels following 4 h after PBM stimulation,^25^ highlighting the transient nature of these responses.

Increased *MMP-3* and *MMP-13* mRNA expression does not necessarily imply a negative effect, as MMPs are secreted as inactive zymogens and require extracellular proteolytical activation to degrade the matrix.^44,45^ Elevated *MMP* expression did not reduce COL II, ACAN, or GAG synthesis, suggesting a disconnect between transcription and enzymatic activity, possibly due to limited MMP activation or compensatory effects of tissue inhibitors (TIMP). Thus, the US may induce MMP production without activating them or may stimulate TIMP synthesis. Gelatin zymography confirmed both inactive and active forms of MMP-2. All stimulation groups increased pro-MMP-2, but only the combined stimulation enhanced its active form. This is particularly interesting, as neither the US nor the PBM alone promoted the MMP-2 activation, while their combination did so in IL-1β-treated chondrocyte cultures. Since MMP-2 is a gelatinase, and MMP-3 and MMP-13 are collagenases, different stimulation modes may elicit distinct responses depending on the MMP subtype.

US and PBM engage distinct mechanotransduction pathways. The US likely activates PIEZO1 ion channels, altering calcium influx,^46–50^ while PBM at 940 nm stimulates TRP channels and mitochondrial cytochrome c oxidase, enhancing metabolism.^51,52^ These differences may explain their divergent anabolic and catabolic effects. Although some studies have investigated US and PBM separately,^53–55^ their combined application remains underexplored. The sequence of application influenced the bioeffects on chondrocytes, particularly *MMP* production, suggesting that each stimulus may activate unique intracellular signaling pathways. However, in general, the combined application did not enhance the bioeffects beyond those achieved by the individual treatments in terms of cartilage matrix synthesis or degradation.

Although this study provides valuable insights into the bioeffects of PBM and US on chondrocytes, several limitations should be acknowledged. The monolayer model does not fully replicate the native cartilage environment. The gene expressions were assessed at a single time point, potentially missing transient responses, and western blot analyses were conducted with limited biological replicates (n = 2). In addition, protein normalization relied on pre-loading quantification and a single housekeeping protein. Future studies should, therefore, incorporate time-course analyses and increased biological replication and total protein normalization approaches, as well as chondrocytes from multiple donors and more physiologically relevant models, to strengthen and validate these findings. Although US experimental setup and parameters were carefully controlled to ensure reproducibility, complex interface effects may still occur,^56,57^ highlighting the need for further optimization and acoustic characterization to fully understand their impact on cellular responses. Future studies may also benefit from finite element method (FEM) simulations to map the spatial distribution of acoustic energy and further refine the characterization of ultrasound exposure conditions.

In conclusion, this study highlights PBM as a promising therapeutic modality for cartilage regeneration, particularly in inflamed environments, where it supports matrix synthesis while minimizing catabolic activation. These findings suggest that PBM could be developed as a noninvasive therapy for early-stage osteoarthritis or cartilage injury, providing a safe strategy to enhance repair even under inflammatory conditions. Although the US also showed encouraging anabolic effects, its proinflammatory potential indicates that careful optimization of dose, timing, and application sequence will be critical for safe clinical use. Importantly, the order of stimulation emerged as a key variable, underscoring that sequencing may influence therapeutic outcomes and should be carefully considered in future translational and clinical studies.

## CONCLUSIONS

This study offers a broad overview of the potential use of PBM and US, both individually or in combination, to improve chondrocyte activity, providing mechanistic insights into their effects on cartilage tissue. PBM consistently promoted cartilage matrix synthesis and preserved a low catabolic profile under both basal and inflammatory conditions, suggesting a potentially favorable biological profile in this context. In contrast, US showed anabolic effects but was also associated with increased expression of inflammatory and degradative markers, highlighting the need for caution and further optimization. Although combining US and PBM altered cellular responses depending on the sequence of application, these effects were not superior to PBM alone. Thus, stimulation order emerges as a key parameter warranting further investigation. Altogether, these findings support the relevance of PBM in cartilage research and emphasize the importance of stimulus-specific and time-sensitive application strategies to optimize noninvasive therapies for joint degeneration.

## METHODS

### Cell culture and seeding

Primary human chondrocytes were commercially sourced from Innoprot (Spain). They were isolated from knee cartilage harvested from a 46-year-old female. Chondrocytes were expanded in basal medium containing 5% (v/v) of fetal bovine serum (FBS), 1% (v/v) penicillin/streptomycin, and 1% (v/v) chondrocyte growth factors at 37 °C and with 5% CO_2_. All culture medium components were purchased from Innoprot (Spain). Cells were trypsinized with 0.25% (v/v) trypsin/EDTA (PAN-Biotech GmbH, Germany) when the cell number reached 90% confluency and subcultured according to the manufacturer's instructions. Passages between 3 and 5 were used in all experiments. Human chondrocytes were seeded at 13 000 cells/ml in 12-well plates, 1 day prior to stimulation. Culture medium was changed every 2 days, and cells were collected for analysis 1 day after the last stimulation session. Chondrocytes were maintained under both basal and proinflammatory conditions. To induce inflammation, chondrocytes were treated with 1 ng/ml of recombinant IL-1β (PeproTech, United States) 3 h after seeding, and fresh IL-1β was added at each medium change.

### Chondrocyte stimulation

**FIG. 9.**
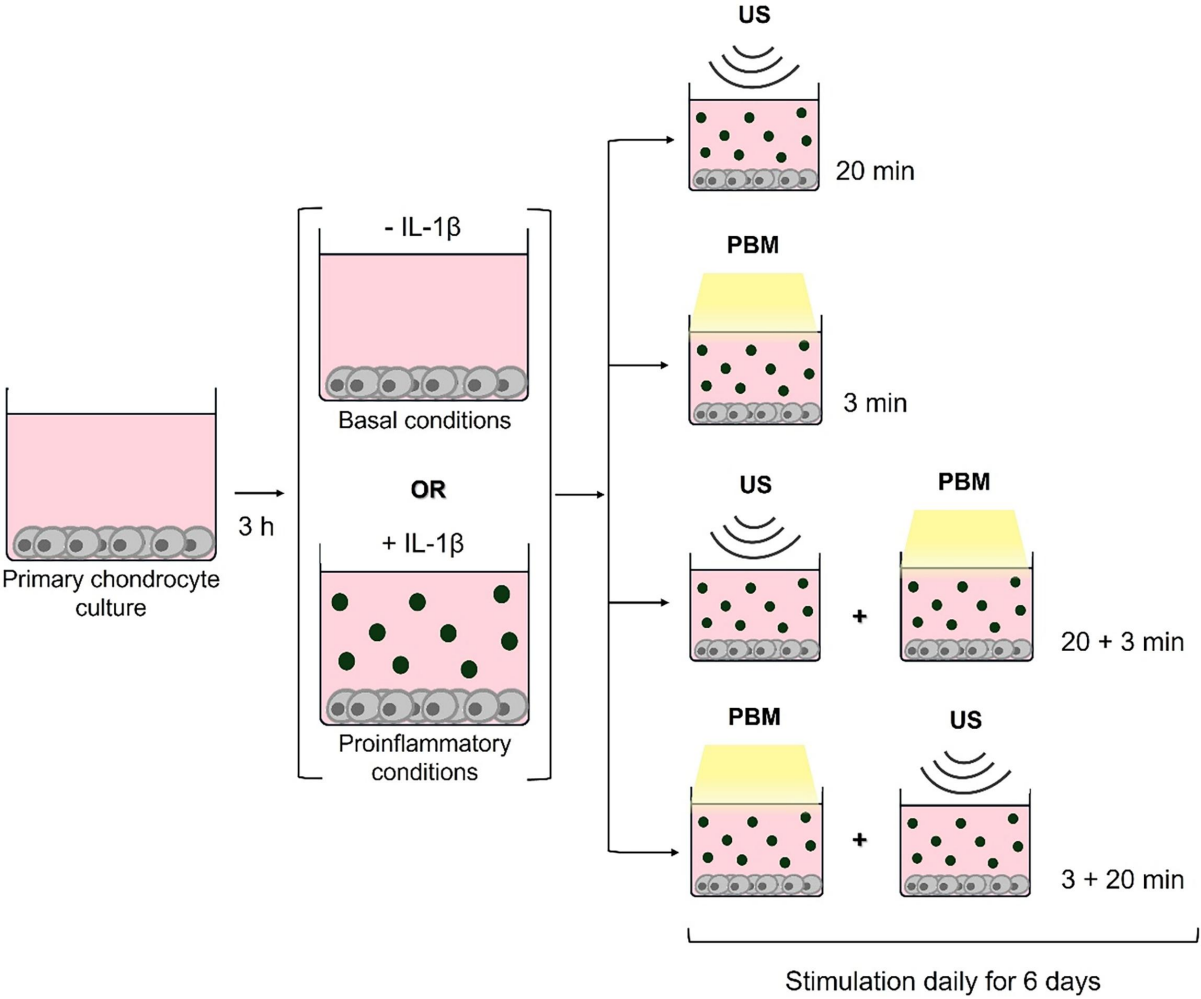
Schematic illustration of experimental study design. After 3 h seeding, human chondrocytes were cultured under basal or proinflammatory conditions (1 ng/ml IL-1β) and stimulated daily for 6 days with US alone, PBM alone, US followed by PBM, and PBM followed by US.

Both IL-1β-free (non-treated) and IL-1β-treated chondrocytes were stimulated with US, PBM, and both. For the combined treatment, two sequences were tested—US followed by PBM (US-PBM), and PBM followed by US (PBM-US)—to assess whether the order of stimulation influenced the biological response, as outlined in the study design schematic shown in Fig. 9. The combined treatments were applied sequentially, with no interval between treatments.

Chondrocytes were stimulated using a custom-built stimulation device developed by our research group,^58^ designed to deliver US through piezoelectric transducers (000084902; PI Ceramic, Germany) made of the ferroelectric soft piezoelectric material PIC255 (modified lead zirconate titanate) and PBM via surface-mounted device of 16 light-emitting diode (LED) arrays (Osram GmbH, Austria), positioned over each well. The piezoelectric transducers were placed in direct contact with the culture medium. The device was connected to a computer via USB, allowing control of the stimulation parameters through custom software developed in-house. For US stimulation, a DC power supply (Uni-Trend Technology Co. Ltd., China) was used to regulate the driving voltage applied to the piezoelectric transducers. Prior to use, the stimulation device was sterilized with UV light for 30 min inside a laminar airflow chamber (Gelaire®, Australia). Before conducting cellular experiments, the acoustic and optical actuation setups were tested and calibrated to ensure accurate intensity delivery at the bottom of the culture wells.

Chondrocytes were stimulated with 250 mW/cm^2^ intensity at 2.00 MHz (pulsed, 1 Hz, 50% duty cycle) for 20 min (for US) and/or with 17 mW/cm^2^, 940 nm (continuous mode) for 3 min (for PBM). The stimulation protocols were defined based on prior work from our group, where the PBM and US parameters and a 6-day experimental timeframe were previously optimized.^22,23^ This timeframe was maintained to ensure methodological consistency and to enable the evaluation of potential synergistic effects under the same experimental conditions. All stimulation procedures were conducted under sterile conditions within a laminar flow cabinet at room temperature. Following stimulation, the 12-well plates were returned to the incubator and maintained at 37 °C with 5% CO_2_. A control group was included in which the customized stimulation device was positioned over the cultured 12-well plate but remained turned off.

### Metabolic activity and cell number

After stimulation, the metabolic activity of IL-1β-free and IL-1β-treated chondrocytes was quantified using the 3-(4,5-dimethylthiazol-2-yl)-5-(3-carboxymethoxyphenyl)-2-(4-sulfophenyl)-2H-tetrazolium (MTS) assay (MTS Cell Proliferation Colorimetric Assay Kit, Abcam, United Kingdom). MTS reagent was added to the culture medium in each well (1:10) and incubated for 3 h at 37 °C with 5% CO_2_. The absorbance was then read at 490 nm using a microplate reader (Biotek Epoch, United States). The absorbance of the blank (culture medium with MTS without cultured cells) was subtracted from all readings.

Cell number was determined using the trypan blue exclusion assay. Human chondrocytes were detached from the well bottom using 0.25% (v/v) trypsin/EDTA for 5 min at 37 °C, followed by centrifugation at 300 × *g* for 5 min. The cell pellet was resuspended in culture medium and mixed with 0.4% (v/v) trypan blue (PAN-Biotech GmbH, Germany). Cell counting was carried out using a Neubauer chamber observed under an inverted microscope (Kern & Sohn GmbH, Germany) at 10× magnification. The MTS absorbance was normalized to the cell number for each condition to ensure accuracy.

### Immunocytochemistry

The deposition of collagen type II (COL II), aggrecan (ACAN), and collagen type I (COL I) on chondrocyte matrix was investigated by immunocytochemistry. Stimulated and non-stimulated chondrocytes, with or without IL-1β treatment, were fixed with 10% (v/v) formalin (Sigma-Aldrich, United States) for 20 min at room temperature. After fixation, chondrocytes cultures were blocked with 50 mM ammonium chloride (NH_4_Cl, ITW Reagents, Italy) for 10 min, permeabilized with 0.1% (w/v) sodium dodecyl sulfate (SDS, Thermo Fisher Scientific, United States) for 10 min, and blocked with 3% bovine serum albumin (w/v) (BSA, NZYtech, Portugal) in phosphate-buffered saline (PBS) for 20 min, at room temperature. Afterwards, chondrocytes were incubated overnight at 4 °C with primary antibodies diluted in 0.1% BSA (w/v) in PBS against COL I (Abbkine, United States, 1:50), COL II (Abbkine, United States, 1:50), and ACAN (Assay Biotechnology, United States, 1:50). Then, chondrocytes were incubated with anti-mouse secondary antibody conjugated with Andy fluor 488 (ABP Biosciences, United States, 10 *μ*g/ml) in 0.1% BSA (w/v) in PBS (for COL II and COL I) or with anti-rabbit secondary antibody conjugated with Alexa Fluor 488 (Abcam, United Kingdom, 1:200) in 0.1% BSA (w/v) in PBS (for ACAN), for 1 h at room temperature, protected from light. Cell nuclei were stained by incubating chondrocyte cultures with 1 *μ*g/ml DAPI (Thermo Fisher Scientific, United States) in PBS for 15 min at room temperature, protected from light. Chondrocytes were observed at the fluorescent microscope Olympus BX51 at 40× magnification, using the appropriate filter settings for Alexa Fluor 488, Andy Fluor 488, and DAPI.

### Alcian blue staining

The synthesis of sulfated glycosaminoglycans (GAGs) by IL-1β-free and IL-1β-treated chondrocytes was determined by the alcian blue staining method, adapted from literature.^59,60^ Stimulated and non-stimulated chondrocytes were fixed with 10% (v/v) formalin for 20 min at room temperature. Cells were then incubated with 1% (w/v) Alcian Blue 8-GX (Sigma-Aldrich, United States) in 0.1 M HCl (pH 1.0) (Sigma-Aldrich, United States) at room temperature in gentle agitation overnight, protected from light. After three PBS washes, chondrocytes were visualized at the fluorescence microscope (Kern & Sohn GmbH, Germany) at a 10× magnification. Afterwards, stained chondrocyte cultures were incubated with 6 M guanidine HCl (Sigma-Aldrich, United States) at room temperature, protected from light, for 3 h in gentle agitation to remove the bound alcian blue. The absorbance of the extracted dye was measured at 630 nm using a microplate reader. The absorbance values were corrected by subtracting the absorbance values of the blank (6 M guanidine HCl).

### Western blot

The protein expression levels of COL II, ACAN, and COL I produced by IL-1β-free and IL-1β-treated chondrocytes were assessed by western blot. After stimulation, total protein was extracted from both non-stimulated and stimulated chondrocytes using the radioimmunoprecipitation assay (RIPA) buffer containing protease inhibitors. Following quantification of total protein concentration in every sample (Pierce BCA Protein Assay kit; Thermo Fisher Scientific, United States), 30 *μ*g of total protein were loaded, separated by 8% sodium dodecyl sulfate–polyacrylamide gel electrophoresis (SDS–PAGE), and transferred onto a methanol-activated polyvinylidene difluoride (PVDF) membrane (Thermo Fisher Scientific, United States). The membranes were blocked with 5% (w/v) nonfat milk in tris-buffered saline containing 0.1% (v/v) Tween 20 (TBS-T) for 1 h at room temperature. After blocking, the membranes were incubated with primary antibodies diluted in TBS-T with 3% (w/v) BSA overnight at 4 °C: COL II (1:200 dilution, sc-518017, Santa Cruz Biotechnology, United States), ACAN (1:500 dilution, L0101, Assay Biotechnology, United States), COL I (1:1000 dilution, Cell Signaling Technology, United States), and β-actin (1:40 000 dilution, Merck, United States). After TBS-T washing, the membranes were incubated with secondary antibodies anti-rabbit or anti-mouse horseradish peroxidase (HRP, Jackson ImmunoResearch, United States) for 90 min at room temperature. The bands were visualized using a chemiluminescent substrate (WesternBright ECL HRP Substrate, Advansta, United States) in a ChemiDoc XRS+ system (Bio-Rad). Band intensity was quantified by ImageJ software (version 1.54g).

### Quantitative PCR

After 6 days of stimulation, total RNA was isolated from IL-1β-free and IL-1β-treated chondrocyte cultures using the Trizol method (Grisp, Portugal), following the manufacturer's instructions. Isolated RNA samples were treated with DNAse I (Thermo Fisher Scientific, United States) and converted to complementary DNA (cDNA) with Xpert cDNA Synthesis Master Mix kit (Grisp, Portugal), according to the manufacturer's protocol. Quantitative PCR (qPCR) was carried out on a CFX Duet Real-Time PCR System (Bio-Rad, United States) using the Xpert Fast SYBR kit (Grisp, Portugal). Supplementary material Table S1 lists the primer sequences, which were synthesized by Metabion and Stabvida. The housekeeping gene *18S* was used to normalize the relative expression levels of *COL I*, *COL II*, *ACAN*, *MMP-3*, *MMP-13*, and *IL-1β*. The relative gene expression was calculated using the 2^−ΔΔCt^ method with control (i.e., untreated cells) set to a value of 1.0.^61^

### Gelatin zymography

One day after the last stimulation session, culture medium was changed, and IL-1β-treated chondrocyte cultures were maintained in serum-free culture medium for two additional days. The conditioned media was then collected and concentrated tenfold using 10 kDa Amicon Ultra centrifugal filters (Millipore) by centrifugation at 4 °C, 6700 × *g*, until the entire volume of conditioned media was reduced accordingly.

MMP-2 secretion and activation in the conditioned media of each experimental group were measured through gelatin zymography. Accordingly, 45 *μ*g of total protein from each sample was loaded and separated by electrophoresis on a 7.5% SDS–PAGE gel containing 4 mg/ml gelatin (Sigma-Aldrich, United States). After electrophoresis, gels were incubated twice with washing buffer for 30 min, and then with incubation buffer for 24 h at 37 °C under gentle shaking. Following incubation, gels were stained with 0.5% (w/v) Coomassie blue (Sigma-Aldrich, United States) in 40% (v/v) methanol/10% (v/v) acetic acid for 1 h at room temperature. Afterwards, gels were destained with 40% (v/v) methanol/10% (v/v) acetic acid at room temperature until clear bands appeared over a dark background. MMP-2 activity was assessed based on the intensity of gelatinolytic bands. Images of the gels were acquired using a camera and analyzed quantitatively with ImageJ software. The band intensities from stimulated IL-1β-treated chondrocytes were normalized to the control (non-stimulated IL-1β-treated chondrocytes). Culture medium without IL-1β incubation served as the negative control for the experiment.

### ELISA

The secretion of MMP-3 by IL-1β-treated chondrocytes was quantified by performing an enzyme-linked immunosorbent assay (ELISA) on the cell culture medium collected for each condition. For this purpose, a human MMP-3 ELISA kit (Elabscience, USA) was used. The assay was performed according to the manufacturer's indications, as well as the preparation of the reagents. Culture medium was diluted according to the manufacturer's protocol. All steps were performed at 37 °C. Standards and samples were incubated in duplicate for 90 min, followed by incubation with the detection antibody for 1 h. After three washing steps, streptavidin-HRP conjugate was added for 30 min, and then the substrate reagent was incubated for 15 min. Afterwards, stop solution was added, and the absorbance was immediately read at 450 nm on the microplate reader. The absorbance of the blank was subtracted from the absorbance of each sample.

### Statistical analysis

Statistical analysis was performed using the 26.0 version of the Statistical Package for the Social Sciences (SPSS, IBM, Chicago, IL) software for Windows. The Shapiro–Wilk test was used to assess the normality of data. For data following normal distribution, Levene's test was first applied to check for equal variances, and, then, one-way ANOVA test followed by Tukey's test was used for multiple comparisons between stimulation groups with control. In the case of data without parametric distribution, first a log transformation was conducted to apply parametric tests for statistical analysis. If data still did not follow normal distribution, the non-parametric Kruskal–Wallis test followed by Tukey's test was applied for multiple comparisons. The p-value was 0.05, and the results were depicted as mean ± standard deviation. The results represent at least two independent biological experiments, each including three technical replicates per condition.

## SUPPLEMENTARY MATERIAL

See the supplementary material for the list of qPCR primer sequences used in this study, as well as the uncropped western blot figures.

## Data Availability

The data that support the findings of this study are available from the corresponding author upon reasonable request.
